# Overexpression of MRPS18-2 in Cancer Cell Lines Results in Appearance of Multinucleated Cells

**Published:** 2013

**Authors:** Z. Shevchuk, M. Y. Yurchenko, S. D. Darekar, I. Holodnuka-Kholodnyuk, V. I. Kashuba, E. V. Kashuba

**Affiliations:** Department of Microbiology, Tumor and Cell Biology (MTC), Karolinska Institutet, Stockholm, 17177, Sweden; Kavetsky Institute of Experimental Pathology, Oncology and Radiobiology of NASU, 45, Vasylkivska str., Kyiv-22, 03022, Ukraine; Kirchenstein Institute of Microbiology and Virology, Riga Stradins University, 5 Ratsupites, Riga, LV-1067, Latvia; Institute of Molecular Biology and Genetics of NASU, 150 Zabolotnogo str., Kyiv-143, 03680, Ukraine

**Keywords:** Mitochondrial ribosomal protein S18-2 (MRPS18-2), multinucleated cells, cancer cell line, cell cycle, RB binding protein

## Abstract

Human mitochondrial ribosomal protein MRPS18-2 (S18-2) is encoded by a cellular gene that is located on the human chromosome 6p21.3. We discovered that overexpression of the S18-2 protein led to immortalization and de-differentiation of primary rat embryonic fibroblasts. Cells showed anchorage-independent growth pattern. Moreover, pathways characteristic for rapidly proliferating cells were upregulated then. It is possible that the S18-2 overexpression induced disturbance in cell cycle regulation. We found that overexpression of S18-2 protein in human cancer cell lines led to an appearance of multinucleated cells in the selected clones.

## INTRODUCTION

Mitochondrial ribosomal protein S18-2 (MRPS18-2, NP_05476, S18-2 in the text) is encoded by a cellular gene located on human chromosome 6p21.3. S18-2 cDNA was cloned after an analysis of the differentially expressed genes in CD34^+^ hematopoietic progenitor cells [1]. The human genome contains three different S18 genes, in contrast to two in *C. elegans* and one in bacteria [2, 3]. The proteins of the S18 family are localized on the surface of the small subunit (28S) of the mammalian mitochondrial ribosome [3]. The function of these proteins is largely unknown.

Recently we shown that overexpression of the human mitochondrial ribosomal protein S18-2 led to immortalization of primary rat embryonic fibroblasts, RE Fs [4]. Cells of the derived cell line named 18IM lost contact inhibition. Moreover, they acquired the ability for anchorage-independent growth in soft agar with a high cloning efficiency (more than 90%). Immortalized 18IM cells expressed the embryonic stem cell markers SSEA-1, Sox2, and Oct4 that were not detected in the original RE Fs*. *Noteworthy, the 18IM cells lost the expression of mesodermal markers like vimentin and smooth-muscle actin. Part of them expressed ecto- and endoderm-specific pan-keratin, ectodermspecific beta-III-tubulin, and mesoderm-specific MHC class II markers; some of the cells differentiated into fat cells in confluent cultures. The 18IM cells produced excessive amounts of pyruvate, suggesting an enhanced ATP synthesis. Moreover, as was shown by microarray analysis and Q-PCR , many genes encoding enzymes that are involved in redox reactions, such as ATP synthases, mitogen activated kinases, and NADH dehydrogenases, are greatly upregulated in the immortalized cells [5]. Pathways of oxidative phosphorylation, ubiquinone biosynthesis, PI3K/AKT signaling, and fatty acid elongation in mitochondria, characteristic for rapidly proliferating cells, were also upregulated in the 18IM cells.

Earlier we had found that S18-2 specifically binds to the retinoblastoma protein, RB [6]. S18-2 competes with E2F1 for the RB binding, thus S18-2 might play a role in the control of G1-S phase transition [7].

In the present work we show that overexpression of S18-2 in the human tumor cell lines MCF7 and KRC /Y leads to the appearance of multinucleated cells.

**Fig. 1:**
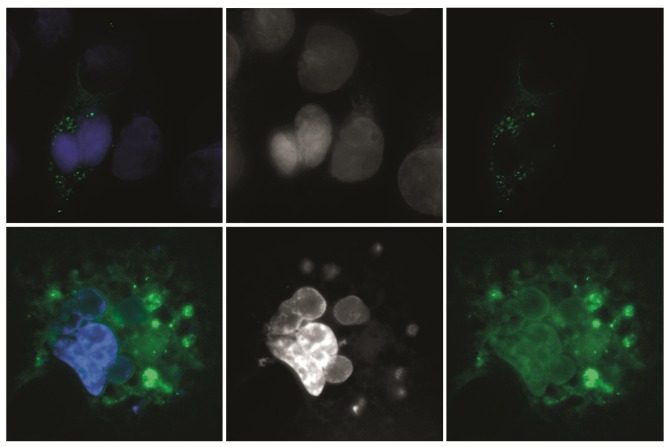
Cellular localization of GFP-S18-2 (panels a–c) and GFP- ΔLANA-S18-2 (panels d–f) in the transfected MCF7 (top row) and KRC/Y (bottom row) cells. Note that in all transfected cells the exogenous S18-2 protein (panels a, c, d, and f) is localized in the cytoplasm

## EXPERIMENTAL PROCEDURES

**Plasmids**

Cloning of S18-2 cDNA into the pEGFPC-1 and pCMV- Tag3A (c-myc-tagged) vectors was described earlier [7]. S18-2 cDNA was also cloned in the pEGFPC-1 vector, coding for a fusion protein GFP-ΔLANA-S18-2, with the first 35 amino acids of LANA, encoded by the human herpes virus 8 (HHV8, Kaposi sarcoma associated herpes virus) at the 5' end. The sequence was verified by direct sequencing, using commercial forward and reverse primers (Stratagene, Santa Clara, CA, USA) and Applied Biosystems sequencer (Perkin Elmer, Wellesley, MA, USA).

**Antibodies**

The following primary antibodies were used: mouse monoclonal anti-c-myc (clone 9E10, Zymed Laboratories Inc., San Francisco, CA, USA), anti-BrdU (Becton Dickinson (BD), San Jose, CA, USA), and anti-actin (Sigma-Aldrich, St. Louis, MO, USA); rabbit anti-S18-2 serum (described in [7]) and anti-MRPS18B (Proteintech Group inc, Chicago, IL, USA), and FITC -conjugated swine anti-rabbit and rabbit anti-mouse (Dako, Glostrup, Denmark).

**Cells, cell culture, immunostaining and imaging**

MCF7 breast carcinoma and KRC /Y renal carcinoma cell lines were cultured at 37°C in a Iscove・Ls medium that contained 10% fetal bovine serum and appropriate antibiotics (penicillin (100 μU /ml) and streptomycin (100 μg/ml)). Periodic staining with Hoechst 33258 (Sigma-Aldrich) monitored the absence of mycoplasma. Prior to transfection experiments, the cells were grown on coverslips. The MCF7 cells were transfected with GFP- and c-myc-tagged constructs (GFP-S18-2 and MT-S18-2, correspondingly), and the KRC /Y cells were transfected with GFP-ΔLANA-S18-2 construct, in parallel with empty vectors, using Lipofectamine and Plus Reagent (Life Technology, Carlsbad, CA), according to the manufacturer’s protocol. Immunostaining and digital image capturing was performed as described elsewhere. Briefly, cells on coverslips were fixed in a 1 : 1 mixture of cold methanol and acetone (–20°C). After rehydration in phosphate-buffered saline (PBS), the cells were stained with antibodies. Hoechst 33258 was added at a concentration of 0.4 μg/ml for DNA staining. Images were captured using DAS microscope Leitz DM RB with a Hamamatsu dual mode cooled charge-coupled device (CC D) camera (C4880; Hamamatsu, Japan).

**Cell cycle analysis by flow cytometry.**

One million of living cells were labeled with bromodeoxyuridine (BrdU, 30 μM) for 30 min at 37°C, trypsinized, collected, and fixed in ethanol (75% in PBS) at 4°C for at least 10 hours. After this, the cells were treated with pepsin (1 mg/ml in 30 mM HCl) for 30 min at 37°C and with 2 M HCl for 15 min. The cell were labeled with anti-BrdU and FITC -conjugated rabbit anti-mouse antibodies and stained with propidium iodide (25 μg/ml in PBS). Сells (1 X 104) were analyzed by flow cytometry, using a FACScan flow cytometer (BD), and the percentage of cells in each phase of the cell cycle was determined with the help of CellQuest software (BD).

**Fig. 2:**
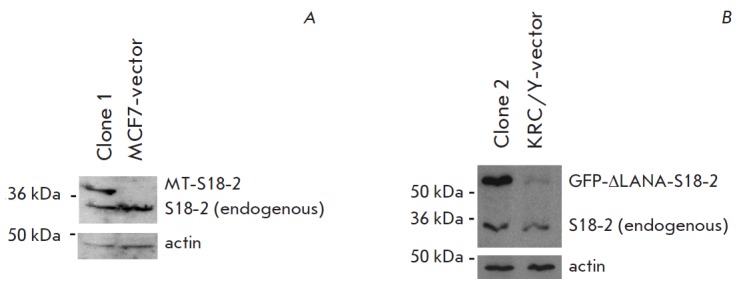
A constitutive expression of S18-2 protein in the MCF7 and KRC/Y cells as compared to vector-transfected cells. The membrane was probed with anti-S18-2 rabbit serum and mouse anti-actin antibodies. Secondary antibodies (sheep anti-rabbit and anti-mouse horse radish peroxidase conjugated, GE-Healthcare, Uppsala, Sweden) and Enhanced chemiluminescence kit (GE-Healthcare) were used to monitor protein bands. A, transfection of MCF7 cells with MT-S18-2 plasmid; B, transfection of KRC/Y cells with GFP-ΔLANA-S18-2 plasmid. Note the expression of endogenous S18-2 protein in all cells

## RESULTS AND DISCUSSION

**Establishment of MCF7 and KRC/Y sublines, expressing S18-2 constitutively**

Cells were grown in 6-well-plates prior transfection. 5 μg of MT-S18-2 or GFP-ΔLANA-S18-2 plasmid was used for transfection. MT-S18-2 and GFP-S18-2 signal was observed in the cytoplasm of the transfected MCF7 cells (*Fig. 1*, the top row, panels *a *and *c*). Earlier we have shown that GFP-S18-2 could be targeted to the nucleus upon cell transformation [4]. In order to achieve a nuclear localization of S18-2, its cDNA was cloned in the GFP-ΔLANA fusion vector that contained the first 35 amino acids of LANA, the HHV8-encoded latent nuclear antigen. It was shown that N-terminus of LANA binds to histones H2A and H2B to tether a HHV8 episome to a chromosome [8]. Despite that, the GFP-ΔLANA-S18-2 fusion protein was observed mainly in the cytoplasm of KRC /Y cells (*Fig. 1*, the lower row, panels *d *and *f*). 48 hours after transfection the cells were transferred to a Petri dish (7.5 cm in diameter) and the selective medium that contained 2 mg/ml G418 was applied. Three weeks later, some of the clones that had survived (12 clones for MT-S18-2 and GFP- ΔLANA-S18-2 plasmids) were isolated and analyzed by Western blotting and immunostaining. Three clones of MCF7 and four clones of KRC /Y expressed exogenous S18-2 at a high level. For the further study, clone 1 of MCF7 and clone 2 of KRC /Y were selected (*Fig. 2A* and *B*). Noteworthy, the endogenous S18-2 was expressed at low levels in both cell lines.

**MT-S18-2 overexpression leads to the appearance of multinucleated cells**

**Fig. 3:**
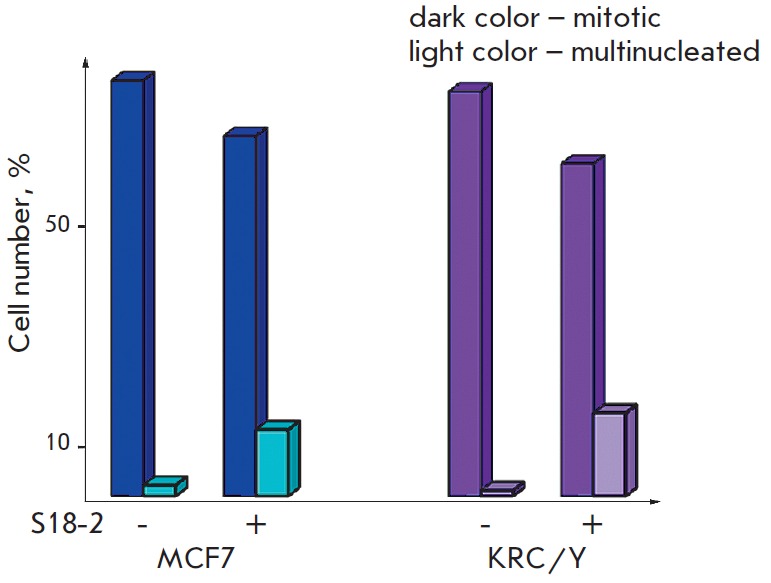
MCF7 and KRC/Y cell cultures, expressing exogenous S18-2 constitutively, show a high proportion of multinucleated cells. Dark-color bars represent the percentage of mitotic cells and light-color bars, percentage of multinucleated cells in the culture

**Fig. 4:**
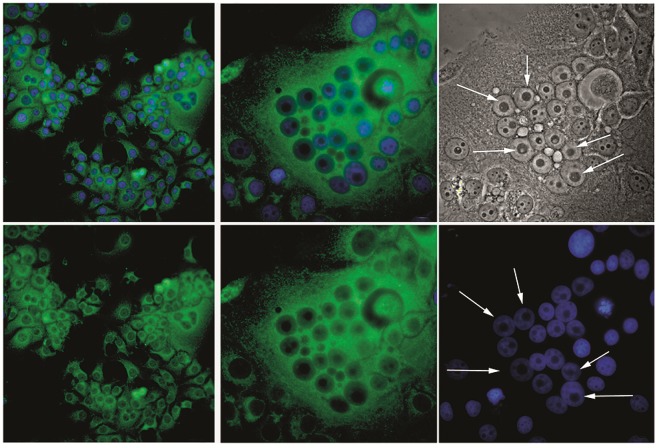
Multinucleated cells in the MCF7 cell culture expressing S18-2 constitutively. The percentage of multinucleated cells is shown in panels a and b (20×). At higher magnification (63×), large single nucleoli in multinucleated cells can be seen (panels c–f). Signal of MT-S18-2 fusion protein stained with anti-c-myc antibody is shown in green; DNA is stained blue

MCF7-clone 1 and KRC /Y-clone 2 cells that expressed S18-2 constitutively, along with vector-transfected cells, were passaged for more than 20 population doublings. We have observed an extremely high frequency of multinucleated cells (*Fig. 3*). Approximately 12% of MCF7 cells and 15% of KRC /Y cells that expressed exogenous S18-2 protein at a high level, were multinucleated. Such cells were observed after sequential freezing and thawing of the culture. Noteworthy, the nucleoli in multinucleated cells were enlarged (*Fig. 4*, panels *c, e,* and *f*), suggesting an enhanced protein synthesis.

**Fig. 5:**
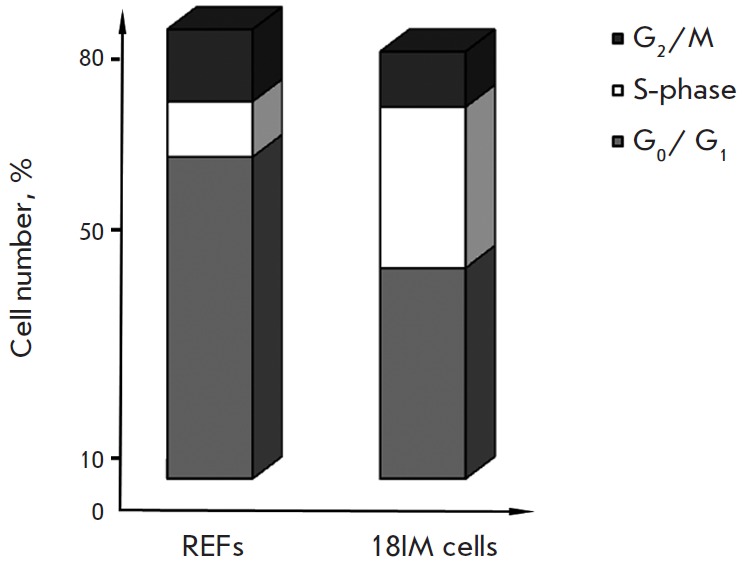
Cell cycle distribution in 18IM cells and control primary fibroblasts (REFs). The percentage of cells in S-phase is clearly elevated in 18IM cells, suggesting the deregulation of cell cycle upon the S18-2 overexpression

In order to investigate a possible mechanism of multinucleated cell formation, we analyzed the cell cycle distribution in 18IM cells. The percentage of cells in S-phase was much higher in 18IM cells than that in RE Fs (*Fig. 5*). A corresponding reduction in the cell number in G1-phase was also observed. A smaller number of cells in G_2_/M phase in 18IM cell culture may suggest that S18-2 protein, when expressed at a high level, leads to elevated transcription/translation, causing impaired mitosis.

How the mitochondrial ribosomal protein S18-2 can influence cell cycle regulation? Actually, it is well known that a crucial role in the G_1_/S checkpoint control is performed by retinoblastoma protein (RB) [9]. A hypo-phosphorylated form of RB binds to E2F1-5 transcription factors and prevents the S-phase entry. Hyper-phosphorylation of RB results in freeing of E2F1-5 and activation of the E2F-dependent transcription. As we mentioned earlier, we have found that S18- 2 protein binds to RB and frees E2F1 from inhibitory complexes with RB, inducing the S-phase [6, 7]. It may explain the increased number of cells in S-phase (*Fig. 5*) and, eventually, the formation of multinucleated cells.

There could be other mechanisms leading to a creation of cells with more than one nucleus. For example, enhanced expression of nucleoporin (Nup153, NP_005115) led to the appearance of multinucleated HeLa cells, due to its binding to the MAD1 protein controlling mitotic spindle-assembly checkpoint [10]. Another example of similar action is overexpression of BCSG1 protein (gamma-synuclein, NP_003078) in breast cancer cell lines, leading to inactivation of BubR1 regulating mitotic checkpoint [11]. Can S18-2 influence the cell division due to its binding to proteins controlling a mitotic pole formation? This question has no answer yet, however. There are more than 70 mitochondrial ribosomal proteins encoded by the human genome (for review see [12]), and their functions are largely unknown. Some studies suggested multiple functions of ribosomal proteins in mammalian cells. It was shown, for example, that MRPS29, one of the proteins of the small mitochondrial ribosome subunit, is not only involved in the ribosome assembly but can also induce apoptosis [13, 14]. The mitochondrial ribosomal protein of large subunit, L41 (MRPL41), can induce G_1_ arrest [15, 16]. To further explore the functions of S18-2, we are currently looking for S18-2 binding proteins.

## CONCLUSIONS

We have shown that overexpression of the mitochondrial ribosomal protein S18-2 in the human cancer cell lines MCF7 and KRC /Y results in the appearance of multinucleated cells. This can be due to enhanced transcription/ translation and, probably, impaired mitosis. Further studies should be performed to analyze this phenomenon.
